# The genome sequence of the Sage Skipper, *Muschampia proto* (Ochsenheimer, 1808) (Lepidoptera: Hesperiidae)

**DOI:** 10.12688/wellcomeopenres.25829.1

**Published:** 2026-01-24

**Authors:** Mattia Menchetti, Joan Carles Hinojosa, Lucas Kaminski, Roger Vila, Charlotte J. Wright, Joana I. Meier, Mark L. Blaxter, Wellcome Sanger Institute Tree of Life Management, Samples and Laboratory team, Wellcome Sanger Institute Scientific Operations: Sequencing Operations, Wellcome Sanger Institute Tree of Life Core Informatics team, Tree of Life Core Informatics collective, Project Psyche Community

**Affiliations:** 1 Institut de Biologia Evolutiva (CSIC-UPF), Barcelona, Catalonia, Spain; 2 Universidade Federal de Alagoas, Maceió, State of Alagoas, Brazil; 3 Tree of Life Programme, Wellcome Sanger Institute, Hinxton, England, UK

**Keywords:** Muschampia proto; Sage Skipper; genome sequence; chromosomal; Lepidoptera

## Abstract

We present a genome assembly from a male specimen of *Muschampia proto* (Sage Skipper; Arthropoda; Insecta; Lepidoptera; Hesperiidae). The assembly contains two haplotypes with total lengths of 542.52 megabases and 541.58 megabases. Most of haplotype 1 (99.67%) is scaffolded into 30 chromosomal pseudomolecules, including the Z sex chromosome. Haplotype 2 was assembled to scaffold level. The mitochondrial genome has also been assembled, with a length of 15.37 kilobases. This work is part of Project Psyche, a collaborative programme generating genomes for European butterflies and moths.

## Species taxonomy

Eukaryota; Opisthokonta; Metazoa; Eumetazoa; Bilateria; Protostomia; Ecdysozoa; Panarthropoda; Arthropoda; Mandibulata; Pancrustacea; Hexapoda; Insecta; Dicondylia; Pterygota; Neoptera; Endopterygota; Amphiesmenoptera; Lepidoptera; Glossata; Neolepidoptera; Heteroneura; Ditrysia; Obtectomera; Hesperioidea; Hesperiidae; Pyrginae; *Muschampia*; *Muschampia proto* (Ochsenheimer, 1808) (NCBI:txid1107688)

## Background

The Sage Skipper, *Muschampia proto* (Ochsenheimer, 1808), is a small butterfly species of the family Hesperiidae found in the Iberian Peninsula, southern France, and North Africa (Morocco and Algeria) ( Hinojosa *et al.*, 2021). The species has xerophilic tendencies and is often found in association with broadly Mediterranean vegetation in open habitats ( García-Barros *et al.*, 2013). It typically occurs at elevations of 200–1 500 m, but has been recorded from 0–1 800 m.

Larvae feed exclusively on *Phlomis* (Lamiaceae). Initially, they construct shelters by binding the edges of leaves with silk and consuming those leaves. As they develop, they move to inflorescences and feed on them ( García-Barros *et al.*, 2013). It overwinters at either the egg or the larval stage ( García-Barros *et al.*, 2013).

*Muschampia proto* is univoltine ( Fernández-Rubio, 1991) and typically flies between April and October ( García-Barros *et al.*, 2013), although adults are rarely observed before June. A partial second generation has been suggested for Catalonia (Spain) ( Vila *et al.*, 2018).

*Muschampia proto* is the type species of the genus, and is part of a complex of cryptic species that started to diverge about 2 million years ago ( Hinojosa *et al.*, 2021): *Muschampia alta*, occurring in southern Italy and the Balkan Peninsula, and *Muschampia proteides*, present in the eastern Europe, the Near East and Iran. These species were, until recently, considered subspecies of *M. proto* ( Hinojosa *et al.*, 2021). For most of its range, *M. proto* shows a low mitochondrial diversity without spatial structure ( Dapporto *et al.*, 2022). Nevertheless, the single specimen sequenced from Algeria is remarkably divergent from other sampled individuals. The species is listed as Least Concern in the IUCN Red List of Europe ( van Swaay *et al.*, 2025).

**Figure 1.**
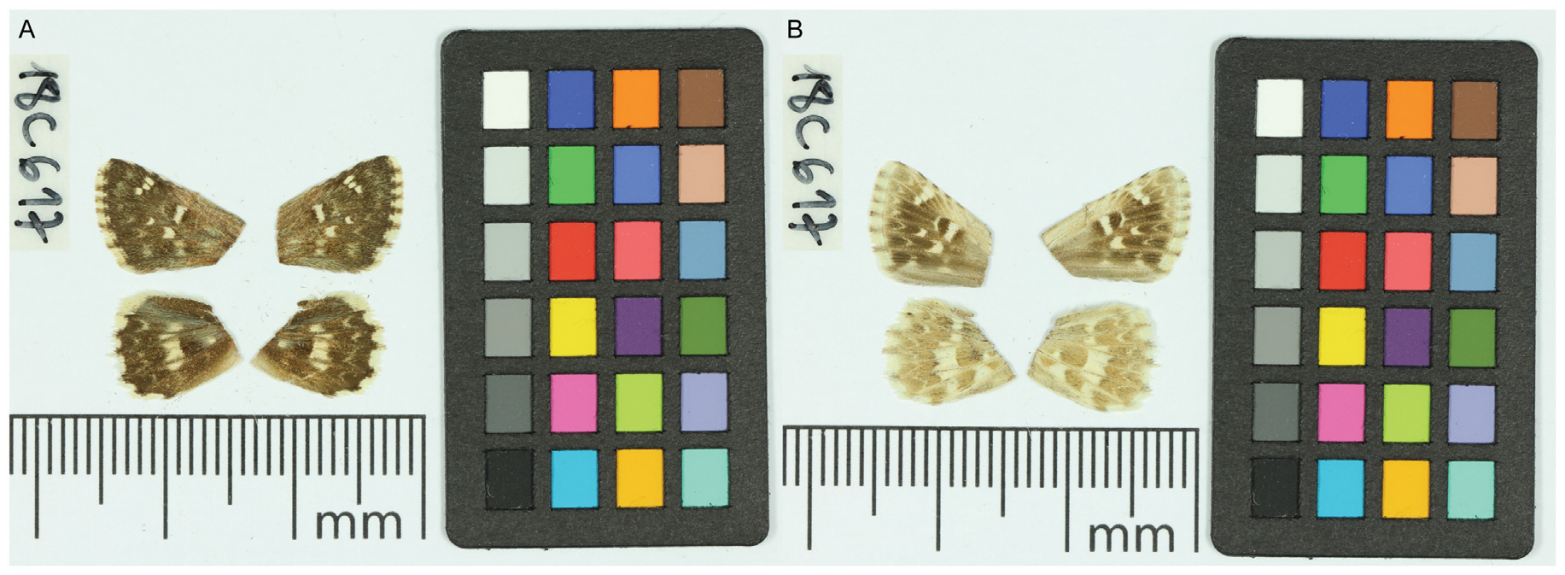
Voucher photographs of the *Muschampia proto* (ilMusProo1) specimen used for genome sequencing.

*Muschampia proto* has a karyotype of 30 chromosomes ( De Lesse, 1970). The first reference genome for *M. proto* presented here offers an valuable resource for investigating the origins of cryptic diversity and the speciation process of *M. proto* and related taxa. The sequence data were derived from a male specimen ( Figure 1) collected from Orcera, Jaén, Andalusia, Spain.

## Methods

### Sample acquisition

The specimen used for genome sequencing was an adult male *Muschampia proto* (specimen ID SAN28000344, ToLID ilMusProo1; Figure 1), collected from Orcera, Jaén, Andalusia, Spain (latitude 38.3091, longitude –2.5825) on 2019-06-06. The specimen was collected by Roger Vila, Mattia Menchetti, Joan Carles Hinojosa, Lucas Kaminski and identified by Roger Vila.

### Nucleic acid extraction

Protocols for high molecular weight (HMW) DNA extraction developed at the Wellcome Sanger Institute (WSI) Tree of Life Core Laboratory are available on protocols.io ( Howard *et al.*, 2025). The ilMusProo1 sample was weighed and triaged to determine the appropriate extraction protocol. Tissue from the whole organism was homogenised by powermashing using a PowerMasher II tissue disruptor.

HMW DNA was extracted in the WSI Scientific Operations core using the Automated MagAttract v2 protocol. DNA was sheared into an average fragment size of 12–20 kb following the Megaruptor®3 for LI PacBio protocol. Sheared DNA was purified by automated SPRI (solid-phase reversible immobilisation). The concentration of the sheared and purified DNA was assessed using a Nanodrop spectrophotometer and Qubit Fluorometer using the Qubit dsDNA High Sensitivity Assay kit. Fragment size distribution was evaluated by running the sample on the FemtoPulse system. For this sample, the final post-shearing DNA had a Qubit concentration of 68.86 ng/μL and a yield of 3 236.42 ng, with a fragment size of 15.2 kb.

### PacBio HiFi library preparation and sequencing

Library preparation and sequencing were performed at the WSI Scientific Operations core. Libraries were prepared using the SMRTbell Prep Kit 3.0 (Pacific Biosciences, California, USA), according to the manufacturer’s instructions. The kit includes reagents for end repair/A-tailing, adapter ligation, post-ligation SMRTbell bead clean-up, and nuclease treatment. Size selection and clean-up were performed using diluted AMPure PB beads (Pacific Biosciences). DNA concentration was quantified using a Qubit Fluorometer v4.0 (ThermoFisher Scientific) and the Qubit 1X dsDNA HS assay kit. Final library fragment size was assessed with the Agilent Femto Pulse Automated Pulsed Field CE Instrument (Agilent Technologies) using the gDNA 55 kb BAC analysis kit. The sample was sequenced on a Revio instrument (Pacific Biosciences). The prepared library was normalised to 2 nM, and 15 μL was used for making complexes. Primers were annealed and polymerases bound to generate circularised complexes, following the manufacturer’s instructions. Complexes were purified using 1.2X SMRTbell beads, then diluted to the Revio loading concentration (200–300 pM) and spiked with a Revio sequencing internal control. The sample was sequenced on a Revio 25M SMRT cell. The SMRT Link software (Pacific Biosciences), a web-based workflow manager, was used to configure and monitor the run and to carry out primary and secondary data analysis.

Specimen details, sequencing platforms, and data yields are summarised in Table 1.

**Table 1.** Specimen and sequencing data for BioProject PRJEB81610.

Platform	PacBio HiFi	Hi-C
**ToLID**	ilMusProo1	ilMusProo1
**Specimen ID**	SAN28000344	SAN28000344
**BioSample (source individual)**	SAMEA115769899	SAMEA115769899
**BioSample (tissue)**	SAMEA115769919	SAMEA115769919
**Tissue**	whole organism	whole organism
**Instrument**	Revio	Illumina NovaSeq X
**Run accessions**	ERR13900447	ERR13907225
**Read count total**	3.88 million	859.80 million
**Base count total**	36.91 Gb	129.83 Gb

### Hi-C

***Sample preparation and crosslinking***

The Hi-C sample was prepared from 20–50 mg of frozen tissue from the whole organism of the ilMusProo1 sample using the Arima-HiC v2 kit (Arima Genomics). Following the manufacturer’s instructions, tissue was fixed and DNA crosslinked using TC buffer to a final formaldehyde concentration of 2%. The tissue was homogenised using the Diagnocine Power Masher-II. Crosslinked DNA was digested with a restriction enzyme master mix, biotinylated, and ligated. Clean-up was performed with SPRISelect beads before library preparation. DNA concentration was measured with the Qubit Fluorometer (Thermo Fisher Scientific) and Qubit HS Assay Kit. The biotinylation percentage was estimated using the Arima-HiC v2 QC beads.

***Hi-C library preparation and sequencing***

Biotinylated DNA constructs were fragmented using a Covaris E220 sonicator and size selected to 400–600 bp using SPRISelect beads. DNA was enriched with Arima-HiC v2 kit Enrichment beads. End repair, A-tailing, and adapter ligation were carried out with the NEBNext Ultra II DNA Library Prep Kit (New England Biolabs), following a modified protocol where library preparation occurs while DNA remains bound to the Enrichment beads. Library amplification was performed using KAPA HiFi HotStart mix and a custom Unique Dual Index (UDI) barcode set (Integrated DNA Technologies). Depending on sample concentration and biotinylation percentage determined at the crosslinking stage, libraries were amplified with 10–16 PCR cycles. Post-PCR clean-up was performed with SPRISelect beads. Libraries were quantified using the AccuClear Ultra High Sensitivity dsDNA Standards Assay Kit (Biotium) and a FLUOstar Omega plate reader (BMG Labtech). Prior to sequencing, libraries were normalised to 10 ng/μL. Normalised libraries were quantified again to create equimolar and/or weighted 2.8 nM pools. Pool concentrations were checked using the Agilent 4200 TapeStation (Agilent) with High Sensitivity D500 reagents before sequencing. Sequencing was performed using paired-end 150 bp reads on the Illumina NovaSeq X.

Specimen details, sequencing platforms, and data yields are summarised in Table 1.

### Genome assembly

Prior to assembly of the PacBio HiFi reads, a database of *k*-mer counts ( *k* = 31) was generated from the filtered reads using FastK. GenomeScope2 ( Ranallo-Benavidez *et al.*, 2020) was used to analyse the *k*-mer frequency distributions, providing estimates of genome size, heterozygosity, and repeat content.

The HiFi reads were assembled using Hifiasm in Hi-C phasing mode ( Cheng *et al.*, 2021; Cheng *et al.*, 2022), producing two haplotypes. Hi-C reads ( Rao *et al.*, 2014) were mapped to the primary contigs using bwa-mem2 ( Vasimuddin *et al.*, 2019). Contigs were further scaffolded with Hi-C data in YaHS ( Zhou *et al.*, 2023), using the --break option for handling potential misassemblies. The scaffolded assemblies were evaluated using Gfastats ( Formenti *et al.*, 2022), BUSCO ( Manni *et al.*, 2021) and MERQURY.FK ( Rhie *et al.*, 2020). The mitochondrial genome was assembled using MitoHiFi ( Uliano-Silva *et al.*, 2023), which runs MitoFinder ( Allio *et al.*, 2020) and uses these annotations to select the final mitochondrial contig and to ensure the general quality of the sequence.

### Assembly curation

The assembly was decontaminated using the Assembly Screen for Cobionts and Contaminants ( ASCC) pipeline. TreeVal was used to generate the flat files and maps for use in curation. Manual curation was conducted primarily in PretextView and HiGlass ( Kerpedjiev *et al.*, 2018). Scaffolds were visually inspected and corrected as described by Howe *et al*. (2021). Manual curation reduced the scaffold count by 18.3%. The curation process is described at https://gitlab.com/wtsi-grit/rapid-curation. PretextSnapshot was used to generate a Hi-C contact map of the final assembly.

### Assembly quality assessment

The Merqury.FK tool ( Rhie *et al.*, 2020), run in a Singularity container ( Kurtzer *et al.*, 2017), was used to evaluate *k*-mer completeness and assembly quality for both haplotypes using the *k*-mer database ( *k* = 31) computed prior to genome assembly. The analysis outputs included assembly QV scores and completeness statistics.

The genome was analysed using the BlobToolKit pipeline, a Nextflow ( Di Tommaso *et al.*, 2017) implementation of the earlier Snakemake version ( Challis *et al.*, 2020). The pipeline aligns PacBio reads using minimap2 ( Li, 2018) and SAMtools ( Danecek *et al.*, 2021) to generate coverage tracks. It runs BUSCO ( Manni *et al.*, 2021) using lineages identified from the NCBI Taxonomy ( Schoch *et al.*, 2020). For the three domain-level lineages, BUSCO genes are aligned to the UniProt Reference Proteomes database ( Bateman *et al.*, 2023) using DIAMOND blastp ( Buchfink *et al.*, 2021). The genome is divided into chunks based on the density of BUSCO genes from the closest taxonomic lineage, and each chunk is aligned to the UniProt Reference Proteomes database with DIAMOND blastx. Sequences without hits are chunked using seqtk and aligned to the NT database with blastn ( Altschul *et al.*, 1990). The BlobToolKit suite consolidates all outputs into a blobdir for visualisation. The BlobToolKit pipeline was developed using nf-core tooling ( Ewels *et al.*, 2020) and MultiQC ( Ewels *et al.*, 2016), with containerisation through Docker ( Merkel, 2014) and Singularity ( Kurtzer *et al.*, 2017).

We painted Merian elements, which represent the 32 ancestral linkage groups in Lepidoptera ( Wright *et al.*, 2024), along chromosomes using lep_busco_painter. Painting used BUSCO gene locations from the lepidoptera_odb10 set ( Kriventseva *et al.*, 2019) and chromosome lengths from NCBI Datasets. Each complete BUSCO (including both single-copy and duplicated BUSCOs) was assigned to a Merian element using a reference database. Positions were coloured and plotted along chromosomes drawn to scale.

## Genome sequence report

### Sequence data

**Figure 2.**
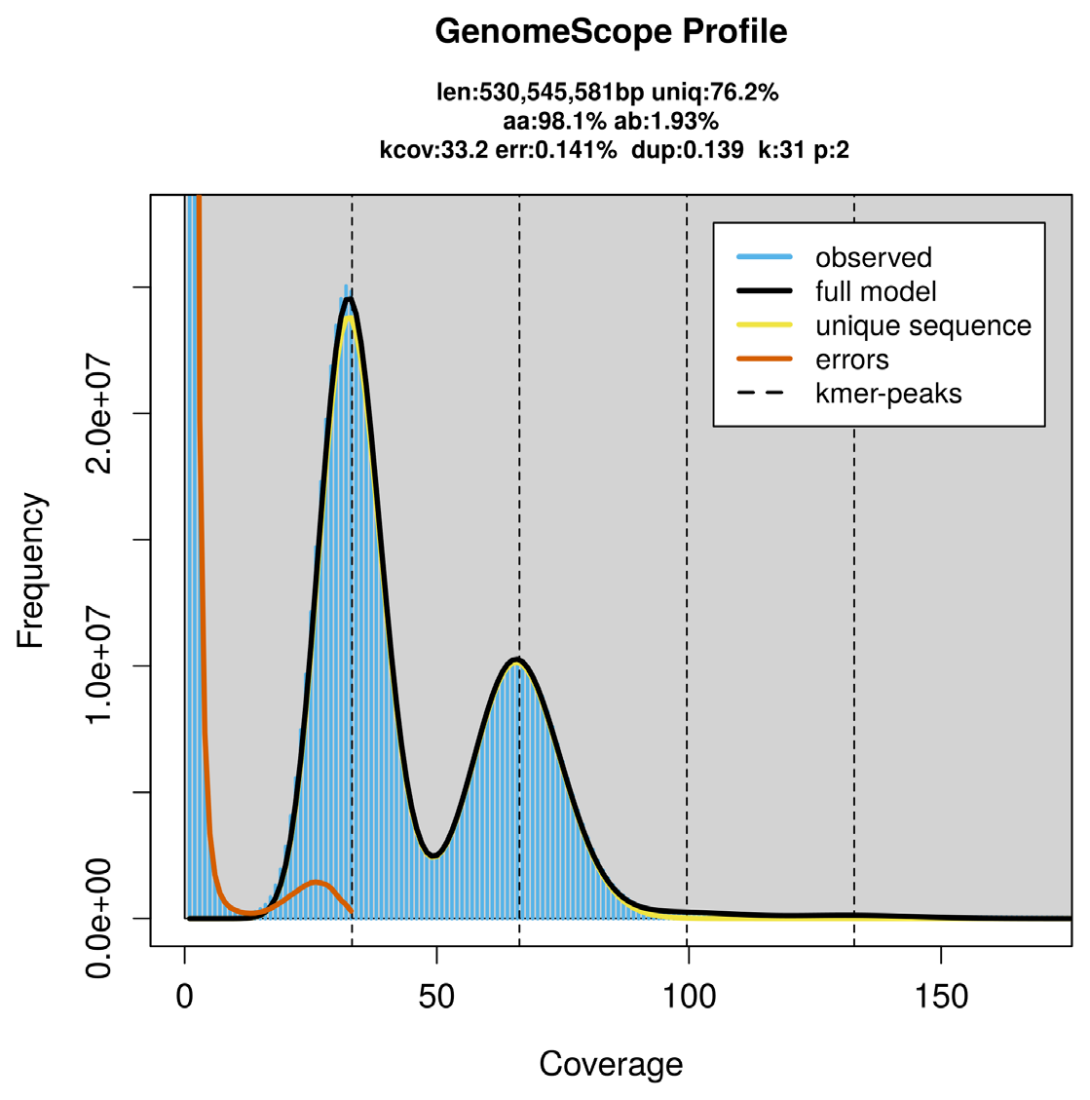
Frequency distribution of *k*-mers generated using GenomeScope2 The plot shows observed and modelled *k*-mer spectra, providing estimates of genome size, heterozygosity, and repeat content based on unassembled sequencing reads.

PacBio sequencing of the *Muschampia proto* specimen generated 36.91 Gb (gigabases) from 3.88 million reads, which were used to assemble the genome. GenomeScope2.0 analysis estimated the haploid genome size at 530.91 Mb, with a heterozygosity of 1.93% and repeat content of 23.81% ( Figure 2). These estimates guided expectations for the assembly. Based on the estimated genome size, the sequencing data provided approximately 66× coverage. Hi-C sequencing produced 129.83 Gb from 859.80 million reads, which were used to scaffold the assembly. Table 1 summarises the specimen and sequencing details.

### Assembly statistics

The genome was assembled into two haplotypes using Hi-C phasing. Haplotype 1 was curated to chromosome level, while haplotype 2 was assembled to scaffold level. The final assembly has a total length of 542.52 Mb in 84 scaffolds, with 88 gaps, and a scaffold N50 of 19.8 Mb ( Table 2).

**Table 2.** Genome assembly statistics.

**Assembly name**	ilMusProo1.hap1.1	ilMusProo1.hap2.1
**Assembly accession**	GCA_964304755.1	GCA_964304745.1
**Assembly level**	chromosome	scaffold
**Span (Mb)**	542.52	541.58
**Number of chromosomes**	30	scaffold-level
**Number of contigs**	172	164
**Contig N50**	7.67 Mb	7.77 Mb
**Number of scaffolds**	84	77
**Scaffold N50**	19.8 Mb	19.78 Mb
**Longest scaffold length (Mb)**	40.53	-
**Sex chromosomes**	Z	-
**Organelles**	Mitochondrion: 15.37 kb	-

**Figure 3.**
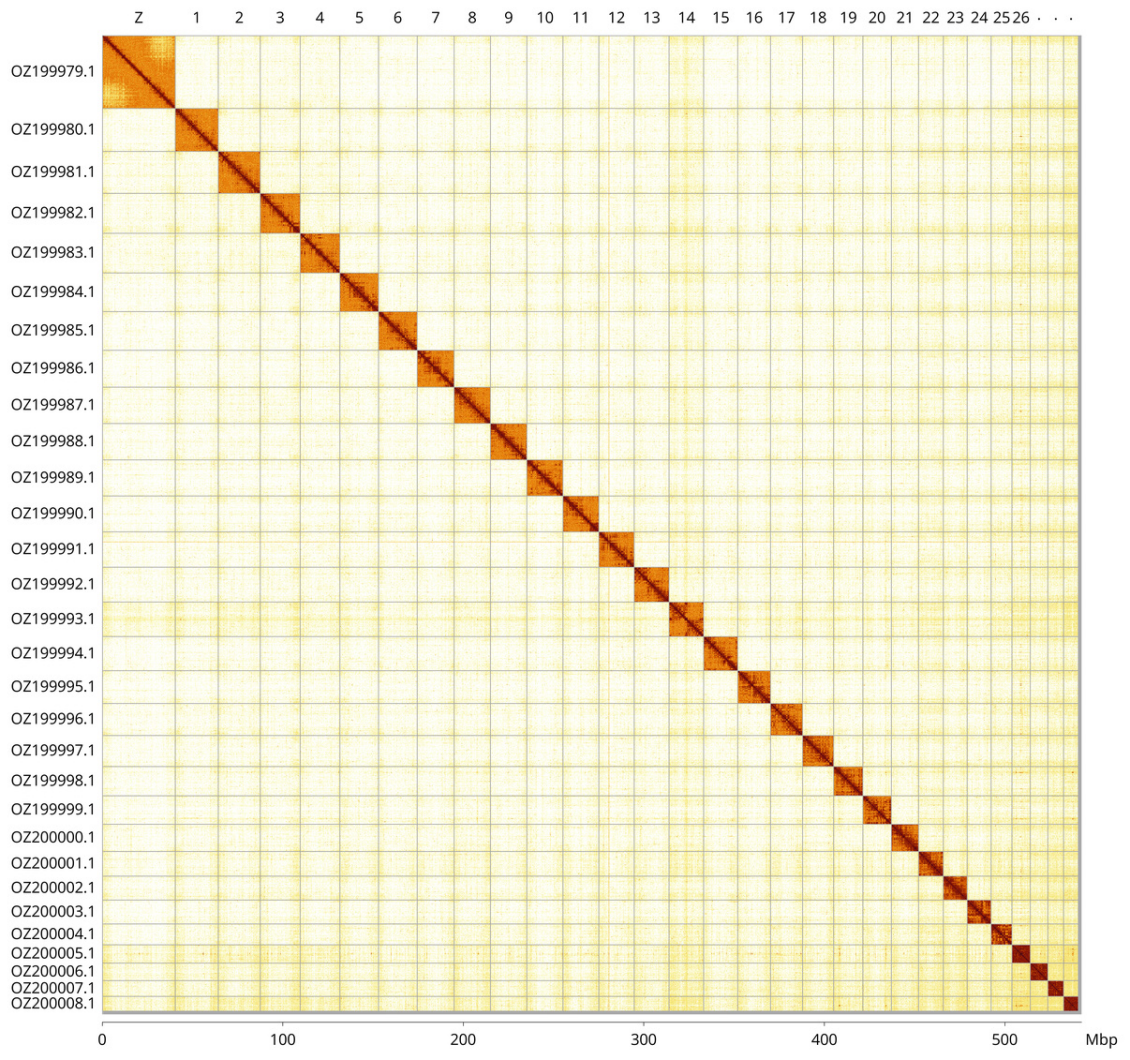
Hi-C contact map of the *Muschampia proto* genome assembly. Assembled chromosomes are shown in order of size and labelled along the axes, with a megabase scale shown below. The plot was generated using PretextSnapshot.

**Figure 4.**
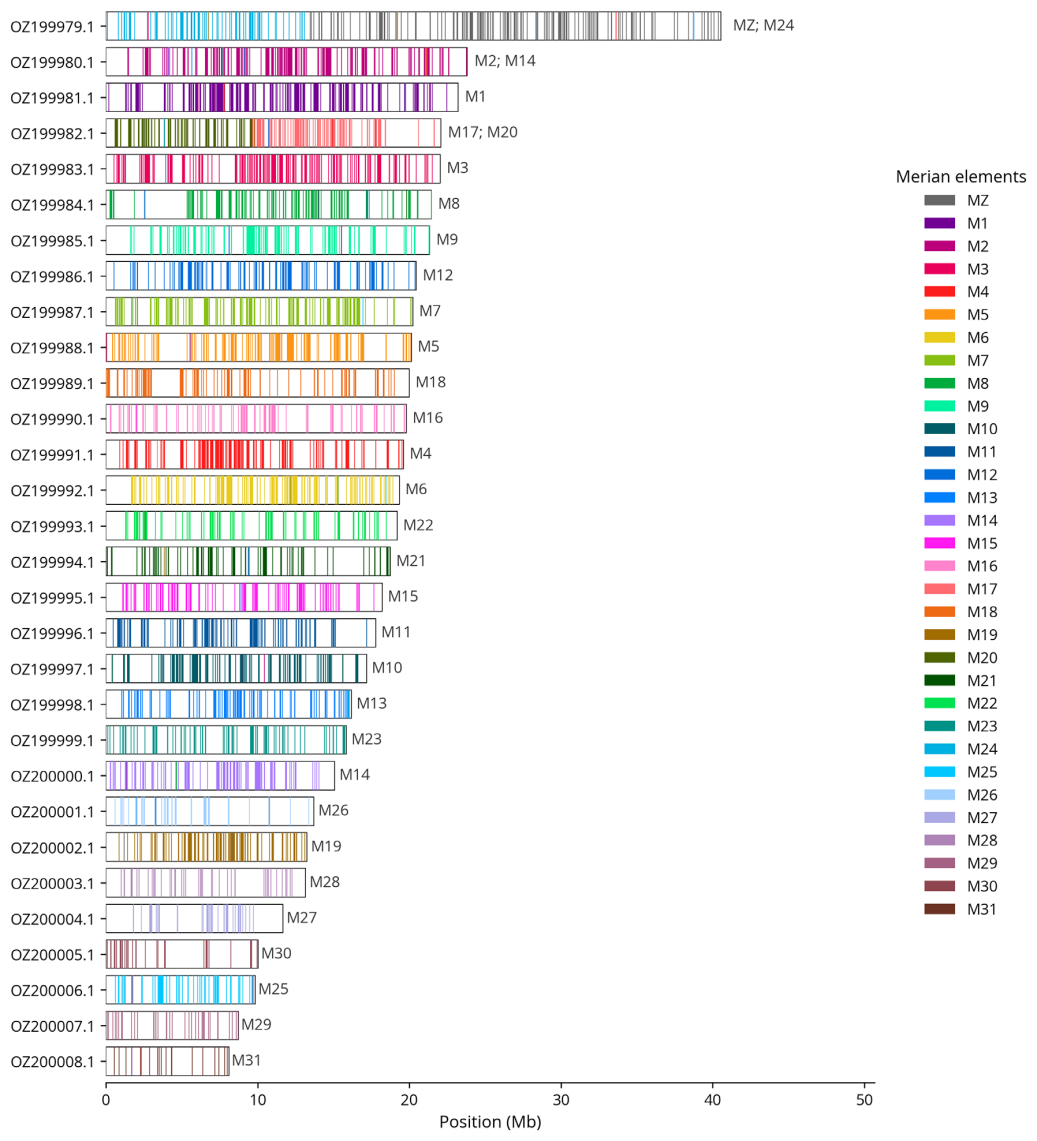
Merian elements painted across chromosomes in the ilMusProo1.hap1.1 assembly of *Muschampia proto*. Chromosomes are drawn to scale, with the positions of orthologues shown as coloured bars. Each orthologue is coloured by the Merian element that it belongs to. All orthologues which could be assigned to Merian elements are shown.

Most of the assembly sequence (99.67%) was assigned to 30 chromosomal-level scaffolds, representing 29 autosomes and the Z sex chromosome. These chromosome-level scaffolds, confirmed by Hi-C data, are named according to size ( Figure 3; Table 3). Chromosome painting with Merian elements illustrates the distribution of orthologues along chromosomes and highlights patterns of chromosomal evolution relative to Lepidopteran ancestral linkage groups ( Figure 4). Chromosome Z was identified by synteny to GCA_905147235.1.

**Table 3.** Chromosomal pseudomolecules in the haplotype 1 genome assembly of *Muschampia proto* ilMusProo1.

INSDC accession	Molecule	Length (Mb)	GC%	Assigned Merian elements
OZ199980.1	1	23.81	35	M14;M2
OZ199981.1	2	23.21	35	M1
OZ199982.1	3	22.09	35	M17;M20
OZ199983.1	4	22.03	35	M3
OZ199984.1	5	21.47	35	M8
OZ199985.1	6	21.34	34.50	M9
OZ199986.1	7	20.46	34.50	M12
OZ199987.1	8	20.23	35	M7
OZ199988.1	9	20.13	35	M5
OZ199989.1	10	20	34.50	M18
OZ199990.1	11	19.80	35	M16
OZ199991.1	12	19.62	35	M4
OZ199992.1	13	19.33	35	M6
OZ199993.1	14	19.21	36	M22
OZ199994.1	15	18.75	35	M21
OZ199995.1	16	18.21	35	M15
OZ199996.1	17	17.78	35.50	M11
OZ199997.1	18	17.18	35	M10
OZ199998.1	19	16.18	35	M13
OZ199999.1	20	15.85	35.50	M23
OZ200000.1	21	15.07	35.50	M14
OZ200001.1	22	13.71	35.50	M26
OZ200002.1	23	13.24	36	M19
OZ200003.1	24	13.15	35.50	M28
OZ200004.1	25	11.66	35.50	M27
OZ200005.1	26	10.02	38.50	M30
OZ200006.1	27	9.83	36.50	M25
OZ200007.1	28	8.73	36.50	M29
OZ200008.1	29	8.12	37	M31
OZ199979.1	Z	40.53	34.50	M24;MZ

The mitochondrial genome was also assembled (length 15.37 kb, OZ200009.1). This sequence is included as a contig in the multifasta file of the genome submission and as a standalone record.

### Assembly quality metrics

**Figure 5.**
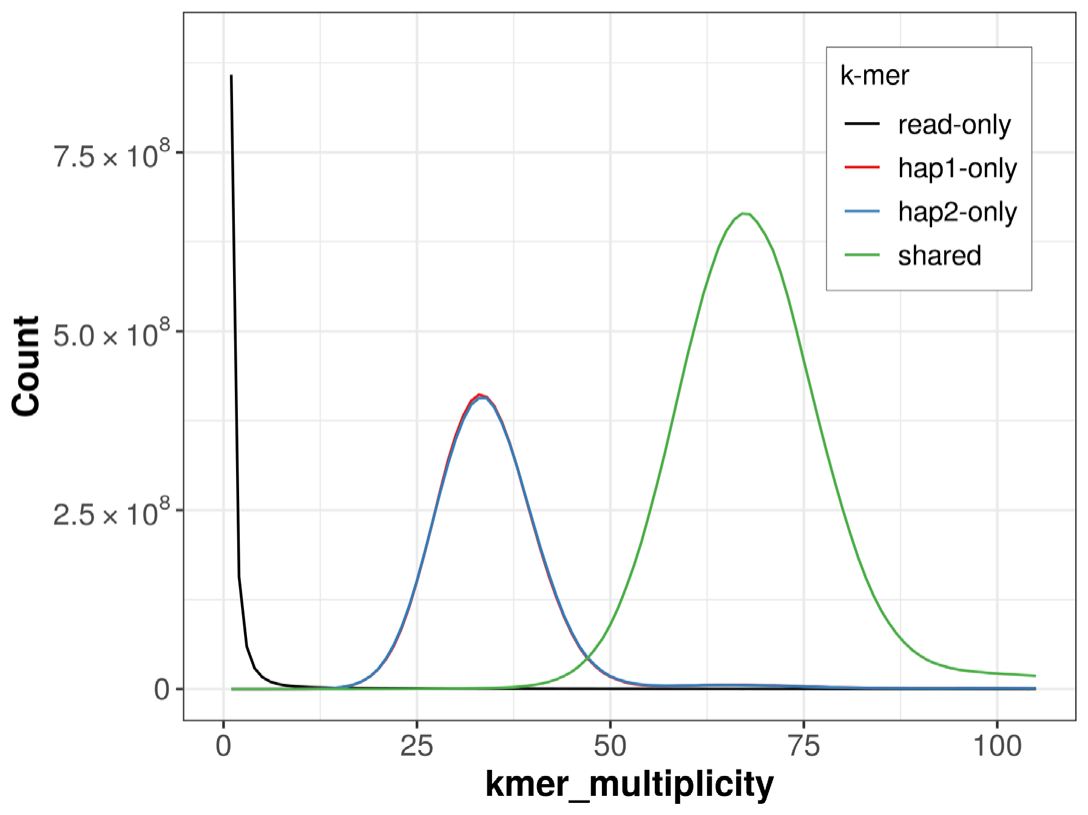
Evaluation of *k*-mer completeness using MerquryFK. This plot illustrates the recovery of *k*-mers from the original read data in the final assemblies. The horizontal axis represents *k*-mer multiplicity, and the vertical axis shows the number of *k*-mers. The black curve represents *k*-mers that appear in the reads but are not assembled. The green curve (the homozygous peak) corresponds to *k*-mers shared by both haplotypes and the red and blue curves (the heterozygous peaks) show *k*-mers found only in one of the haplotypes.

**Figure 6.**
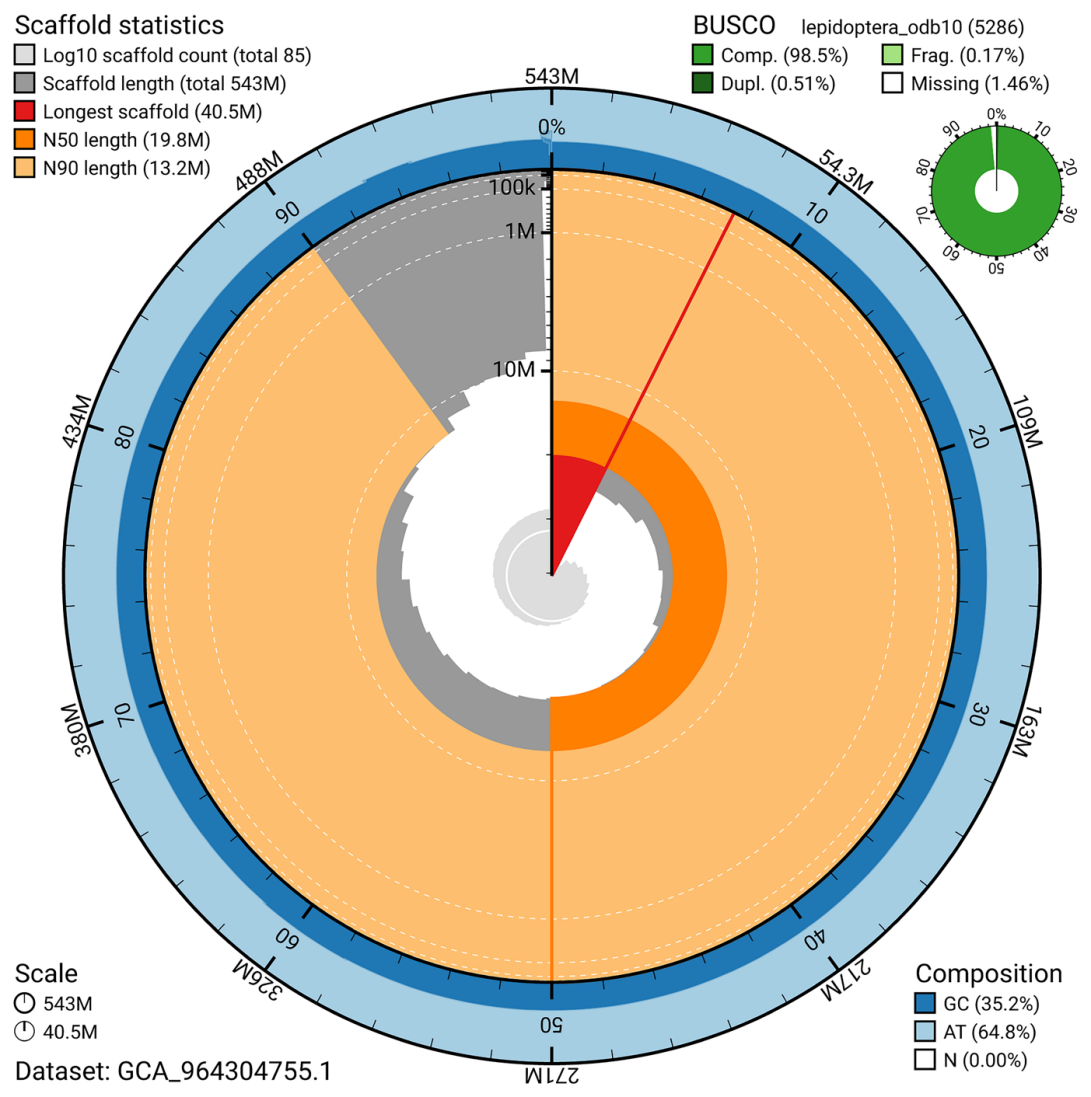
Assembly metrics for ilMusProo1.hap1.1. The BlobToolKit snail plot provides an overview of assembly metrics and BUSCO gene completeness. The circumference represents the length of the whole genome sequence, and the main plot is divided into 1,000 bins around the circumference. The outermost blue tracks display the distribution of GC, AT, and N percentages across the bins. Scaffolds are arranged clockwise from longest to shortest and are depicted in dark grey. The longest scaffold is indicated by the red arc, and the deeper orange and pale orange arcs represent the N50 and N90 lengths. A light grey spiral at the centre shows the cumulative scaffold count on a logarithmic scale. A summary of complete, fragmented, duplicated, and missing BUSCO genes in the set is presented at the top right. An interactive version of this figure can be accessed on the BlobToolKit viewer.

**Figure 7.**
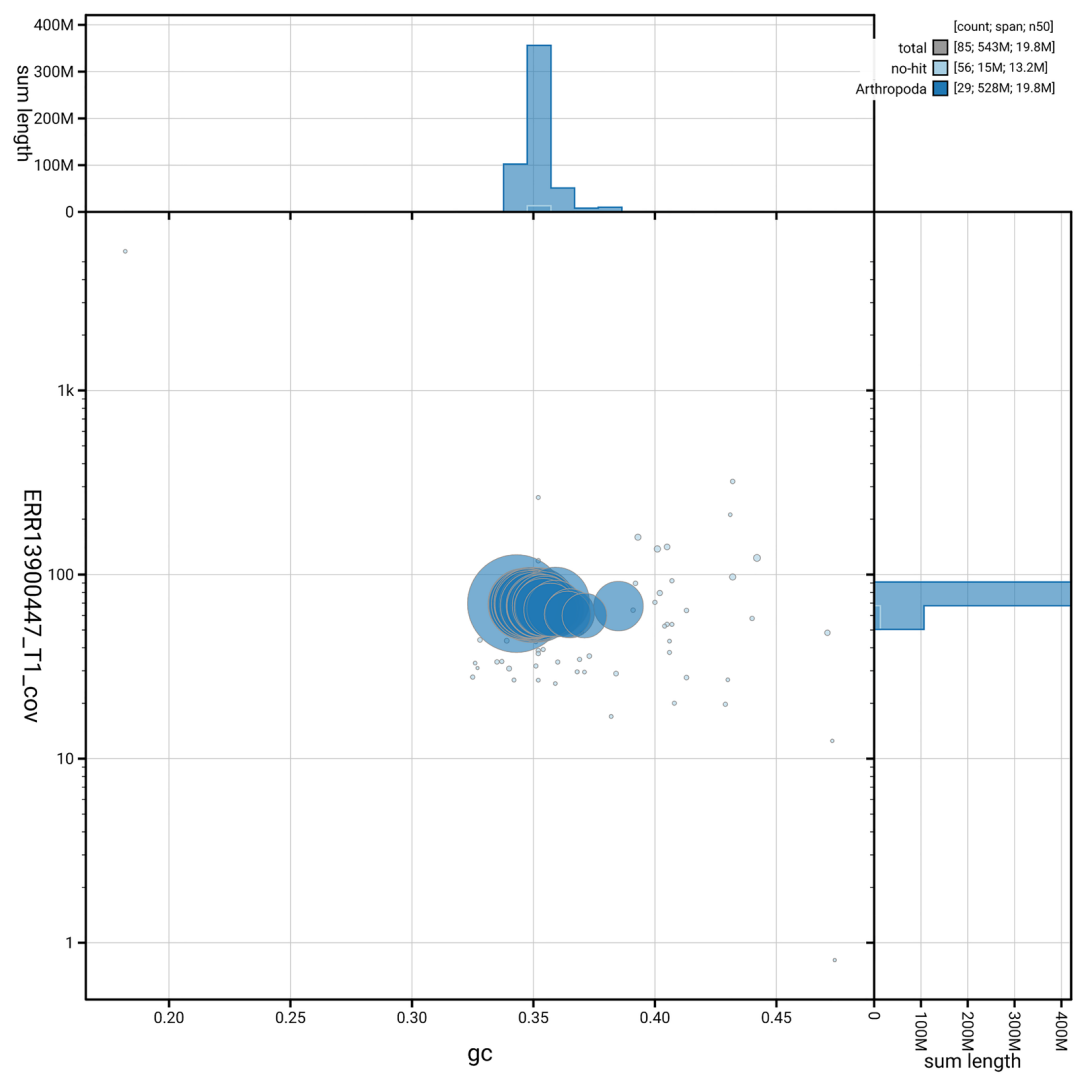
BlobToolKit GC-coverage plot for ilMusProo1.hap1.1. Blob plot showing sequence coverage (vertical axis) and GC content (horizontal axis). The circles represent scaffolds, with the size proportional to scaffold length and the colour representing phylum membership. The histograms along the axes display the total length of sequences distributed across different levels of coverage and GC content. An interactive version of this figure is available on the BlobToolKit viewer.

For haplotype 1, the estimated QV is 65.0, and for haplotype 2, 65.2. When the two haplotypes are combined, the assembly achieves an estimated QV of 65.1. The *k*-mer completeness is 68.93% for haplotype 1, 68.89% for haplotype 2, and 99.78% for the combined haplotypes ( Figure 5). BUSCO analysis using the lepidoptera_odb10 reference set ( *n* = 5 286) identified 98.5% of the expected gene set (single = 98.0%, duplicated = 0.5%) in haplotype 1. For haplotype 2, BUSCO analysis identified 98.6% of the expected gene set (single = 98.1%, duplicated = 0.4%). The snail plot in Figure 6 summarises the scaffold length distribution and other assembly statistics for haplotype 1. The blob plot in Figure 7 shows the distribution of scaffolds by GC proportion and coverage for haplotype 1.

Table 4 lists the assembly metric benchmarks adapted from Rhie *et al.* (2021) the Earth BioGenome Project Report on Assembly Standards September 2024. The EBP metric, calculated for the haplotype 1, is **6.C.Q65**, meeting the recommended reference standard.

**Table 4.** Earth Biogenome Project summary metrics for the *Muschampia proto* assembly.

Measure	Value	Benchmark
EBP summary (haplotype 1)	6.C.Q65	6.C.Q40
Contig N50 length	7.67 Mb	≥ 1 Mb
Scaffold N50 length	19.80 Mb	= chromosome N50
Consensus quality (QV)	Haplotype 1: 65.0; haplotype 2: 65.2; combined: 65.1	≥ 40
*k*-mer completeness	Haplotype 1: 68.93%; Haplotype 2: 68.89%; combined: 99.78%	≥ 95%
BUSCO	C:98.5% [S:98.0%; D:0.5%]; F:0.2%; M:1.3%; n:5 286	S > 90%; D < 5%
Percentage of assembly assigned to chromosomes	99.67%	≥ 90%

**Notes:** EBP summary uses log10(Contig N50); chromosome-level (C) or log10(Scaffold N50); Q (Merqury QV). BUSCO: C=complete; S=single-copy; D=duplicated; F=fragmented; M=missing; n=orthologues

### Wellcome Sanger Institute – Legal and Governance

The materials that have contributed to this genome note have been supplied by a Tree of Life collaborator. The Wellcome Sanger Institute employs a process whereby due diligence is carried out proportionate to the nature of the materials themselves, and the circumstances under which they have been/are to be collected and provided for use. The purpose of this is to address and mitigate any potential legal and/or ethical implications of receipt and use of the materials as part of the research project, and to ensure that in doing so, we align with best practice wherever possible. The overarching areas of consideration are:

- Ethical review of provenance and sourcing of the material
- Legality of collection, transfer and use (national and international).

Each transfer of samples is undertaken according to a Research Collaboration Agreement or Material Transfer Agreement entered into by the Tree of Life collaborator, Genome Research Limited (operating as the Wellcome Sanger Institute), and in some circumstances, other Tree of Life collaborators.

## Data Availability

European Nucleotide Archive: Muschampia proto. Accession number PRJEB81610. The genome sequence is released openly for reuse. The *Muschampia proto* genome sequencing initiative is part of the Sanger Institute Tree of Life Programme (PRJEB43745) and Project Psyche (PRJEB71705). All raw sequence data and the assembly have been deposited in INSDC databases. The genome will be annotated using available RNA-Seq data and presented through Ensembl at the European Bioinformatics Institute. Raw data and assembly accession identifiers are reported in Table 1 and Table 2. Pipelines used for genome assembly at the WSI Tree of Life are available at https://pipelines.tol.sanger.ac.uk/pipelines. Table 5 lists software versions used in this study.

## Author information

Contributors are listed at the following links:

- Members of the Wellcome Sanger Institute Tree of Life Management, Samples and Laboratory team
- Members of Wellcome Sanger Institute Scientific Operations – Sequencing Operations
- Members of the Wellcome Sanger Institute Tree of Life Core Informatics team
- Members of the Tree of Life Core Informatics collective
- Members of the Project Psyche Community
