# Supplementary material for: The genome sequence of the Sage Skipper, Muschampia proto (Ochsenheimer, 1808) (Lepidoptera: Hesperiidae)
Source: Wellcome Open Res. 2026 Jan 24;11:74. [Version 1] doi: 10.12688/wellcomeopenres.25829.1 (PMC13049433; doi:10.12688/wellcomeopenres.25829.1)
Supplement: Peer review history

# Reviewer report: Reviewer response for version 1

**DOI:** 10.21956/wellcomeopenres.28430.r151364

**Published:** 2026-04-14

**Authors:** Yukun Sun

**Affiliations:** 1 The Field Museum, Chicago, Illinois, USA

This Data Note presents a reference genome for the Sage Skipper, *Muschampia proto*, from a male specimen collected in Spain. The paper reports two haplotypes. Haplotype 1 is scaffolded into 30 chromosomal pseudomolecules, including the Z chromosome. Haplotype 2 stays at the scaffold level. The mitochondrial genome is also assembled. The reason for creating the dataset is clear. The note links this genome to future work on cryptic diversity and speciation in *Muschampia* and related taxa.

 The methods are described well for this article type. The paper gives the specimen details, DNA extraction steps, sequencing methods, assembly workflow, curation steps, and software versions. The accession numbers and software table also make the workflow easier to follow and repeat. On this basis, the study looks technically sound.

 The data are presented in a clear and usable way. The paper gives the BioProject, sample, run, and assembly accessions, and it states that the raw data and assemblies are deposited in INSDC databases for reuse. The main quality values are also easy to find. Haplotype 1 has a span of 542.52 Mb, a scaffold N50 of 19.8 Mb, 99.67% of the sequence assigned to chromosomes, BUSCO completeness of 98.5%, and QV 65.0. The Hi-C contact map also supports chromosome-scale scaffolding.

 I do have some points that should be addressed in revision.

 Points that should be addressed:

1. The coverage value should be checked and explained. The paper reports 36.91 Gb of PacBio HiFi data and a haploid genome size estimate of 530.91 Mb. These numbers give about 69.5x coverage, not 66x. If the authors used filtered reads or another denominator, the paper should say so. This is a basic assembly number, so it should be exact.
2. The Z chromosome assignment needs one more sentence. The specimen is male, and the paper shows a Z chromosome in haplotype 1, while haplotype 2 stays at the scaffold level. The authors should state whether the second Z homolog is present in haplotype 2 but not at the chromosome level, or whether another explanation accounts for this pattern. It would also help to name the reference genome used for the synteny-based Z call, not only give the accession.
3. The contamination screen needs a result statement. The methods state that the assembly was decontaminated with ASCC, but the paper does not indicate whether contaminant or co-biont scaffolds were found, removed, or kept as separate records. One short sentence on the final outcome would clarify the QC process.

Are sufficient details of methods and materials provided to allow replication by others?

Yes

Is the rationale for creating the dataset(s) clearly described?

Yes

Are the datasets clearly presented in a useable and accessible format?

Yes

Are the protocols appropriate and is the work technically sound?

Yes

Reviewer Expertise:

Bioinformatics, genomics, phylogenomics, genome assembly, and computational biology

I confirm that I have read this submission and believe that I have an appropriate level of expertise to confirm that it is of an acceptable scientific standard, however I have significant reservations, as outlined above.

# Reviewer report: Reviewer response for version 1

**DOI:** 10.21956/wellcomeopenres.28430.r151361

**Published:** 2026-04-08

**Authors:** Vadim Pisarenco

**Affiliations:** 1 Universitat of Barcelona, Barcelona, Spain

The authors present a high-quality, chromosome-level genome assembly of the Sage Skipper ( *Muschampia proto*), which will serve as a valuable genomic resource for studying cryptic diversity and speciation in *Muschampia* genus. 

 The article is well-written, with an elaborate introduction on the *M. proto *biology and its scientific interest. The methods are clearly described and detailed enough to allow replication by others. I have some minor suggestions, outlined below:

 1- Standardise the nomenclature of the species to *Muschampia proto *or *M. proto*.

 - *M. proto* is now used occasionally throughout the manuscript.

 2- In "Genome sequence report" - "Sequence data" section

 - "GenomeScope2.0 analysis estimated the haploid genome size at 530.91 Mb" -> According to Figure 2, it should be 530.55 Mb.

 3- In "Genome sequence report" - "Assembly quality metrics" section 

 - BUSCO percentages sum 100% in Table 4, but do not in Figure 6 (100,13) due to % of Missing BUSCOs (1.46% in Figure 6 vs 1.3% in Table 4).

 4- Typo in "Background section"

 - an valuable -> a valuable

 All references have been checked.

Are sufficient details of methods and materials provided to allow replication by others?

Yes

Is the rationale for creating the dataset(s) clearly described?

Yes

Are the datasets clearly presented in a useable and accessible format?

Yes

Are the protocols appropriate and is the work technically sound?

Yes

Reviewer Expertise:

Evolutionary and comparative genomics

I confirm that I have read this submission and believe that I have an appropriate level of expertise to confirm that it is of an acceptable scientific standard.

# Reviewer report: Reviewer response for version 1

**DOI:** 10.21956/wellcomeopenres.28430.r151365

**Published:** 2026-04-03

**Authors:** Matthew Merkin

**Affiliations:** 1 University of Liverpool, Liverpool, UK

The authors present a chromosome-level assembly of the Sage Skipper (Muschampia proto) genome. A single male sample was sequenced using both PacBio HiFI and Hi-C methods for long read assembly and scaffolding. A BUSCO completeness of 98.5% and EBP score of 6.C.Q65 suggest that this assembly is of high quality and sufficient for use as a reference genome.

 The background is well researched, providing useful information on such a poorly reported species, as well as suggesting uses of the genome. However, I would like to have seen some reference to Project Psyche in the intro to explain why this genome was assembled.

 I am also not convinced by scaffold OZ199980.1 being a fusion between M2 and M14 based on Figure 4. Instead, M14 appears to be a separate scaffold (OZ200000.1).

Are sufficient details of methods and materials provided to allow replication by others?

Yes

Is the rationale for creating the dataset(s) clearly described?

Yes

Are the datasets clearly presented in a useable and accessible format?

Yes

Are the protocols appropriate and is the work technically sound?

Yes

Reviewer Expertise:

genome assembly, entomology, museomics, population genetics

I confirm that I have read this submission and believe that I have an appropriate level of expertise to confirm that it is of an acceptable scientific standard.

# Reviewer report: Reviewer response for version 1

**DOI:** 10.21956/wellcomeopenres.28430.r147894

**Published:** 2026-03-09

**Authors:** Temitope Opeyemi Oriowo

**Affiliations:** 1 Centre for Molecular Biodiversity Research, Leibniz Institute for the Analysis of Biodiveristy Change, Museum Koenig Bonn, Bonn, North Rhine Westphalia, Germany

This is a well-written article about the genome assembly of *Muschampia proto*. In particular, I appreciate the detailed introduction to the species and understand that it would be a valuable addition to future research into the diversity of this cryptic genus.

 The methods section is well-detailed, and while I was initially concerned about the lack of software versions, I am satisfied to see them displayed in a table subsequently.

 Assembly Statistics indicate a well-assembled haplotype-resolved genome, and benchmarking against the 'gold standard' was a nice touch. The reports of heterozygosity and repeat content would be nice to put in the context of other butterfly species. This would help us to better understand whether it is as expected or unique.

 All the data, including the sequencing and the scripts, is accessible under the correct project numbers.

 All references have been checked and validated.

Are sufficient details of methods and materials provided to allow replication by others?

Yes

Is the rationale for creating the dataset(s) clearly described?

Yes

Are the datasets clearly presented in a useable and accessible format?

Yes

Are the protocols appropriate and is the work technically sound?

Yes

Reviewer Expertise:

Comparative and Population genomics

I confirm that I have read this submission and believe that I have an appropriate level of expertise to confirm that it is of an acceptable scientific standard.
